# The burden of cycling-related trauma to the orthopaedic and trauma department of a level 1 trauma hospital in Adelaide, South Australia

**DOI:** 10.1186/s13018-021-02242-7

**Published:** 2021-02-10

**Authors:** John M. Abrahams, Christopher Sagar, Mark Rickman

**Affiliations:** 1 Department of Orthopaedics and Trauma, Royal Adelaide Hospital, Adelaide, South Australia Australia; 2 Discipline of Orthopaedics and Trauma, Faculty of Health Sciences, Adelaide Medical School, The University of Adelaide, Adelaide, South Australia Australia; 3 Centre for Orthopaedic & Trauma Research (COTR), Adelaide Medical School, The University of Adelaide, Adelaide, South Australia Australia

**Keywords:** Orthopaedic surgery, Trauma, Cycling, Bicycle, Level one trauma, Public safety, Road safety

## Abstract

**Background:**

With the fourth largest metropolitan population density, motor vehicle drivers in Adelaide, South Australia, record the most number of motor vehicle insurance claims in Australia. Previous studies have shown a rise in cycling-related emergency department presentations from 2005 to 2010. There is no specific data available specifically related to South Australia. Our institution is the largest level 1 trauma centre in South Australia and Northern Territory and has a local geographic pool of the central metropolitan region of Adelaide.

The aims of this study were to establish the demographics of cycling-related presentations to our institution that involved the admission of a patient under the Orthopaedic and Trauma service. Secondary aims were to investigate whether there were any common variables among these presentations that could be modified to prevent or reduce the morbidity of cycling-related trauma.

**Methods:**

A prospective study was performed at our institution from 1 March 2018 until 31 December 2019 of all inpatient admissions under the Orthopaedics and Trauma department, where the patient was injured as a cyclist. We collated patient-reported information about the accident and their cycling habits.

**Results:**

One hundred and ten patients were included in the study. One hundred and thirty-one injuries were recorded, requiring 89 surgical procedures. Eighty were upper limb injuries (61%), 49 were lower limb (37%), and 2 injuries occurred in either the spine or ribs. The most common reason for the accident was excessive cyclist speed.

**Conclusions:**

The majority of cyclists admitted to our unit with orthopaedic injuries were male patients who assessed themselves as experienced riders, and yet still were involved in accidents that resulted predominantly from episodes of poor judgement. Speed is a common and avoidable factor involved in the presentation of orthopaedic-related trauma to the public system. Involvement of other vehicles was relatively uncommon, as was poor weather; upper limb injuries predominate in this group.

## Introduction

Cycling is both a means of transport and a recreational activity. The popularity of cycling has increased over the last decade owing to the relative financial benefits, ease of accessibility and convenience, and the perceived health benefits [1–3]. Since the coronavirus pandemic, it has increased significantly across the globe, and as electric assisted bikes have improved, even more people are taking to cycling [4]. In Australia, it is compulsory to wear a helmet whilst cycling, and safety lights are necessary in the dark. Disc brakes have become almost standard, adding safety especially in wet conditions compared to standard brake shoes; in addition to this, new automotive technology such as radar-guided automatic braking technology, and blind spot assist may potentially prevent or result in less severe accidents [5].

A recent retrospective review of cycling-related trauma to emergency departments in Queensland recommended improvement of cycling lanes, development of ergonomic gear, and introduction of new laws to keep distances from cyclists [6, 7]. Previous Australian studies have shown a rise in overall emergency department presentations from 2005/2006 to 2009/2010 [8]. Although previous studies have examined injury patterns of cycling fatalities [9–12], there is no data available specifically related to non-fatal orthopaedic presentations in South Australia.

Our institution is the largest Level 1 trauma centre in South Australia and Northern Territory and has a local geographic pool of the central metropolitan region of Adelaide. In line with new social campaigns that have been established that encourage cycling, exercise, and road safety, it is of interest to establish locally how much of a burden cycling-related trauma has on society, both from a financial perspective, but also a personnel perspective and what possible impact this has on the health care system.

The aims of this study were to establish the demographics of cycling-related presentations to our institution that involved the admission of a patient under the Orthopaedic and Trauma service. Secondary aims were to investigate whether there were any common variables among these presentations that could be modified to prevent or reduce the morbidity of cycling-related trauma.

## Methods

A prospective study was performed at our institution from March 2018 to December 2019 of all inpatient admissions under the Orthopaedics and Trauma department, where the patient was a cyclist. Patients were asked to voluntarily participate and complete a survey about the demographics surrounding the accident. This study was approved by the ethics committee of our institution.

Inclusion criteria:

- All patients aged over 18 that were managed by the Orthopaedic and Trauma department within the study time period
- Any patient that was riding a push bike at the time of the accident

Exclusion criteria:

- Any patient that was not able to complete the questionnaire due to language barrier
- Any patient that refused to participate

The single-page questionnaire consisted of the following questions:

- Age
- Gender
- How much riding do you do each week?
- Was the accident on a commute ride?
- Basic mechanism
- Speed of bike
- Surface
- Weather
- Incline
- Speed of the other vehicle if involved
- Type/make of bike
- Protective gear worn
- What could have prevented this accident?
- How much less speed would you have needed to avoid this accident?
- Did you have any substances on board?

A diagnosis was recorded for each patient prospectively and the time of inpatient stay as well as whether or not they required surgery. Any complications as a result from treatment were also documented. The financial burden of the combined presentations was calculated as follows:

- Length of stay
- Cost of surgery
- Time away from employment

## Results

There were 110 patients that agreed to participate and matched the inclusion criteria. No eligible patients refused to participate.

The median age was 46, range 18 to 90 years of age. Ninety-five males and fifteen females were included in the cohort. Ninety-four (85%) of the patients considered themselves to be regular cyclists. Thirty-four (31%) of the accidents took place during a regular commute.

The basic mechanism has been grouped and is described in Table 1. The most common reason for the accident was excessive speed. The median self-reported speed of the bike at the time of the accident was 30 km/h (range 0 to 55). For the 56 cyclists that listed speed as a cause, the median suggested drop in speed that would have been required to avoid the accident was 20 km/h (range 5 to 40). Wet weather was reported as a cause in 12 *twelve cases. Twenty-five patients stated that either they were unsure, listed a non-specific cause, or stated it was not possible to avoid the accident.

**Table 1:** Self-reported reason of the accident. Note the total number of causes is greater than the number of accidents because a number of patients listed multiple reasons

Reason	Number
Too fast	56
Road surface	16
Mechanical issue	15
Driver of vehicle	14
Fatigue	12
Should not have tried to do stunt	10
Too slow	8
Substances on board	3
Unsure/other/not avoidable	25

Nine patients stated that they had taken substances prior to cycling, with three having had methamphetamines, four having alcohol, one having diazepam, and one having a combination of alcohol and marijuana. Three of these patients were not wearing any protective gear. No other patients reported that they were not wearing protective gear. Sixteen of the presentations were reported to be as a result of an accident with another vehicle, where the vehicle may have been at fault. In eight cases, the road surface was blamed for the accident.

The most common bike used at the time of presentation was a road/racing bike, in 46 cases (Table 2).

**Table 2:** Self-reported type of bike used during accident

Type of bike	Number
Racing/road bike	46
Mountain bike	31
BMX bike	9
Electrical assisted bike	7
Not documented	17

One hundred and thirty-one injuries were recorded, requiring 89 procedures. Eighty injuries were in the upper limb (61%), 49 injuries occurred in the lower limb (37%), and 2 injuries occurred in either the spine or ribs. The most commonly injured region in the upper limb was the clavicle or shoulder (34 injuries). The most commonly injured region in the lower limb was the tibia (14 injuries) (Table 3).

**Table 3:** Reported injuries for all 110 study participants. Injuries are listed in the order of commonality

Injury	Number
Forearm fracture	29
Clavicle injury	25
Tibia fracture	14
Pelvic fracture	12
Ankle fracture	11
Neck of femur fracture	8
Hand fracture	8
Soft tissue Injury	6
Scapular fracture	6
Carpal injury	3
Rotator cuff injury	2
Foot injury/fracture	2
Spinal injury	1
Rib fracture	1
ACL/meniscal injury	1
Proximal humerus fracture	1
Femur fracture excluding neck of femur	1

## Discussion

With the fourth largest metropolitan population density, Adelaide drivers record the most number of motor vehicle insurance claims per 100,000 in the nation [13]. Between 2007 and 2018, cycling traffic in the Adelaide Central Business District was observed to increase by 56%. Despite this increase in cycling traffic, the number of fatalities/serious injuries and minor injuries have largely remained constant [14].

In this study, the majority of patients considered themselves to be experienced cyclists (101 of 110), with regular and long distance riding per week (mean 118 km/week, min 2 km, max 700 km). Despite this, only 16 of 110 presentations involved another vehicle. A large number were due to poor judgement that included either misjudging a corner, performing “wheelies” or “jumps”, or going down an excessively steep descent. Weather was not a commonly listed reason for the accident, with only twelve cases being attributed to wet weather—this may reflect the generally dry Adelaide climate. Interestingly, the majority of injuries encountered were in the upper limb, compared to the normal distribution of orthopaedic trauma presentations to our institution that would favour the lower limb.

There are a number of limitations to this study. First, there may be presentations that were missed despite the prospective nature of the study. The cases however were cross-checked with the department trauma database, and so omissions should be very few. This study only includes patients presenting to a single institution, and indeed to the Orthopaedic and Trauma department. There were likely patients within the same time frame that sustained non-orthopaedic injuries requiring inpatient care under other specialties. Patients were also not recruited if they had died prior to referral to the Orthopaedic and Trauma service. Therefore, the figures reported in this study are likely to be under-representation of the burden on the overall medical system. The self-reported causes and circumstances that have been documented are those that are reported by patients themselves, and not of independent observers: a patient-reported injury mechanism (PRIM). This was done using a descriptive questionnaire, which has not been specifically validated but was designed to give us as much information as possible regarding both the rider and the accident. This has some inherent inaccuracy, in addition to which some of the accident reasons may not be completely accurate due to patient fear of being implicated in responsibility.

This study is the first to report data for the local geographic location. Environmental causes were implicated in only twenty cases (18%) of presentations, being weather and the road surface. Despite a commonly held belief that shared cycling lanes alongside roads pose a significant risk to cyclists, only 15% of our cases involved another vehicle. Adelaide has a high volume of cyclists due to its climate and terrain, and especially around the time of the Tour Down Under cycling race. This low rate of vehicle involvement is likely a testament to local safety awareness, well-planned cycling lanes, and the effects of large volumes of cyclists on a regular basis. A similar study by De Rome et al, performed in the Australian Capital Territory also similarly found that cycling related accidents were less likely to occur in dedicated in on-road cycle lanes than in other cycling environments [8].

In contrast, cyclist insight revealed that in half of the cases, speed was the major avoidable factor that led to the accident. In 91% of cases, the perceived cause was due to rider factors rather than external causes. This highlights that even in an experience rider cohort, public health awareness campaigns should address risk-taking behaviours. An emphasis on speed, use of illegal substances, and fatigue would be of benefit. Although males also predominate, we do not have the figures to assess if this is representative of the cycling population or not.

Despite the emergence of an increasing number of electrical assisted bicycles, both owned and rented, there were only seven cyclists in our study that were riding e-bikes. In other cities, the use of electrical assisted bicycles has been associated with an increased number of accidents [15].

The amount of time and staff required to assess each patient is significant, from the multiple medical staff required for the initial assessment in the resuscitation pathway, to the perioperative resources utilised and the aftercare follow-up associated with multiple outpatient visitations. Given that cycling-related injuries are mainly avoidable, this represents a significant resource use that could potentially be used elsewhere.

In a similar retrospective study performed in New Zealand, Singh et al. found that the promotion of cycling within the community was not supported by a similar level of improvement in infrastructure which may have contributed to a 16.8% year on year increase in the presentation of cycling-related trauma to their institution [16].

Other countries have implemented a road trauma registry. The results of the French road trauma registry was investigated by Amoros et al. who described that the severity of accidents that took place in towns where less severe and less likely to involve a motor vehicle compared to accidents outside of towns [17]. The implantation of a road trauma registry with a correlation to clinical outcomes would be of benefit in Australia.

## Conclusion

The majority of cyclists admitted to our unit with orthopaedic injuries were male patients who assessed themselves as experienced riders. Speed was reported by riders to be the most commonly avoidable factor involved in the presentation of orthopaedic-related trauma to the public system. Involvement of other vehicles was not often reported. Future public health awareness campaigns should look to address these findings.

## Data Availability

N/A
